# Multiple revision anterior cruciate ligament reconstruction: not the best but still good

**DOI:** 10.1007/s00167-022-07197-8

**Published:** 2022-10-12

**Authors:** Riccardo D’Ambrosi, Amit Meena, Akshya Raj, Nicola Ursino, Matteo Formica, Mirco Herbort, Christian Fink

**Affiliations:** 1 IRCCS Orthopedic Institute Galeazzi, Via Galeazzi 4, 20161 Milan, Italy; 2 Dipartimento Di Scienze Biomediche Per La Salute, Università Degli Studi Di Milano, Milan, Italy; 3 Gelenkpunkt – Sports and Joint Surgery, FIFA Medical Centre of Excellence, Innsbruck, Austria; 4 Research Unit for Orthopaedic Sports Medicine and Injury Prevention (OSMI), Private University for Health Sciences, Medical Informatics and Technology, Innsbruck, Austria; 5 Central Institute of Orthopaedics, Vardhman Mahavir Medical College and Safdarjung Hospital, New Delhi, India; 6 UOC Clinica Ortopedica IRCCS Policlinico San Martino, Genova, Italy; 7 Dipartimento di Scienze Chirurgiche e Diagnostiche Integrate (DISC), Università degli Studi di Genova, Genova, Germany; 8 Orthopadische Chirurgie Munchen, Munchen, Germany

**Keywords:** Anterior cruciate ligament, Revision, Re-revision, Multiple, Injury, A systematic review

## Abstract

**Purpose:**

Given the paucity of literature on the re-revision of ACL, the current study was undertaken. The purpose of this systematic review was to synthesise and qualitatively assess the currently available evidence in the literature regarding the re-revision of ACL reconstruction (rrACLR).

**Methods:**

A systematic review was conducted based on the PRISMA guidelines. The following search terms were used in the title, abstract and keywords fields: “ACL” or “anterior cruciate ligament” AND “revision” or “multiple” or “repeat”. The outcome data extracted from the studies were the Lysholm score, Subjective IKDC, Marx Score, Tegner, Marx Score, KOOS score, radiological changes and the rate of return to sports. Complications, failures and/or revision surgery were also analysed.

**Results:**

The cohort consisted of 295 patients [191 (64.7%) men and 104 (35.3%) women] with a mean age of 29.9 ± 2.8 years (range 14–58 years) from 10 studies. The mean postoperative follow-up (reported in all studies except one) was 66.9 ± 44.7 months (range 13–230.4 months). Associated injuries were 103 (34.9%) medial meniscus tears, 57 (19.3%) lateral meniscus tears, 14 (4.7%) combined medial plus lateral meniscus tears, 11 (3.7%) meniscal tears (not specified), 252 (85.4%) cartilage lesions, 6 (2.0%) medial collateral ligament injury and 2 (0.7%) lateral collateral ligament injuries. In 47 (15.9%) patients an extra-articular plasty was performed for the anterolateral ligament. In all studies that reported pre- and post-operative IKDC (subjective and objective) and Lysholm score, there was a significant improvement compared to the pre-operative value (*p* < 0.05). At the final follow-up, laxity measured with KT-1000 was found to be 2.2 ± 0.6 mm. 31 (10.5%) out of 295 patients returned to their pre-injury activity level. A total of 19 (6.4%) re-ruptures were found, while only 4 (1.4%) complications (all minors) were reported, out of which 2 (0.7%) were superficial infections, 1 (0.3%) cyclops lesion and 1 (0.3%) flexion loss.

**Conclusion:**

Multiple revisions of anterior cruciate ligament reconstruction allow acceptable clinical results and a good degree of knee stability with a low rate of subsequent new re-ruptures but the possibility of regaining pre-injury sports activity is poor; whenever possible, it is preferred to revise the ligament in one stage. This surgery remains a challenge for orthopaedic surgeons and many doubts persist regarding the ideal grafts, additional extra-articular procedures and techniques to use.

**Level of evidence:**

IV.

**Study registration:**

PROSPERO**-**CRD42022352164 (https://www.crd.york.ac.uk/prospero/).

**Supplementary Information:**

The online version contains supplementary material available at 10.1007/s00167-022-07197-8.

## Introduction

ACL reconstructions are one of the most commonly performed arthroscopic reconstructive procedures and this has only been estimated to increase over the next few years until 2025 by some computer-based predictive models [20]. Understandably, such increasing ACL surgeries will lead to a greater number of failures, which will in turn cause the revision surgery rates to rise. The rate of revision after primary ACL reconstruction has been reported to be between 4 and 13% and is now with the increasing burden of revision [21].

The revision of a failed ACL reconstruction can be particularly challenging considering that the surgeon needs to account for previous bony tunnels and their positions, the widening of previous tunnels, the presence of implants from previous surgery, post-surgical adhesions, bone loss and concurrent injuries to other structures of the knee that might impact the outcome of the current revision surgery [21]. These factors also have a bearing on whether one performs a single-staged or two-staged revision surgery. Preoperative tunnel widening has been reported as an indication of a two-staged revision ACL reconstruction [15, 16]. However, a recent cohort study has reported excellent results with a single-stage strategy for revision ACL reconstruction [26].

The superiority of one- or two-staged procedures over the other is yet to be proven, with most of the available evidence comparing two-staged and single-staged strategies being retrospective [26].

Thus, it can be stated that these challenges are only being compounded in the setting of a re-revision of a failed revision ACL reconstruction. Another important consideration facing surgeons is the choice of graft. In the setting of multiple revisions, the grafts from the ipsilateral limb might be unavailable and the patient cannot allow for any surgery on the uninjured limb the contralateral uninjured limb to be subjected to surgical procedure for graft harvesting [8, 29].

The revision of failed ACL reconstruction has been well studied in the literature. Although with time, the techniques have improved and the outcomes after a revision surgery have seen progress, the results after revision ACL surgery have still been reported to be inferior compared to primary ACL surgery in terms of patient-reported outcomes, clinical knee function scores and the incidence of degenerative changes [29]. Following revision anterior cruciate ligament reconstructions, failure rates up to 30% have been reported in the literature and the outcomes following revision anterior cruciate ligament reconstructions have been inferior to primary reconstructions, with failure rates up to 4 times higher in the former compared to primary ACL reconstruction [5].

These failed revisions require re-revision surgeries. Hence, there is an urgent need for a better understanding of re-revision surgery but there are lacunae in contemporary literature concerning outcomes following a re-revision [17, 28]. With the demonstrable rise of ACL reconstructions and more patients wanting to remain active in their pre-injury sports activities, there is going to be an inevitable rise in the number of re-revisions with the risk of repeated injuries [17, 28].

The purpose of this systematic review was to synthesise and qualitatively assess the currently available evidence in the literature regarding the re-revision of ACL reconstruction (rrACLR).

It has been hypothesised that re-revision surgery can provide good knee stability with satisfactory clinical results.

## Materials and methods

The current systematic review was performed following the Preferred Reporting Items for Systematic Reviews and Meta-Analyses (PRISMA) guidelines and is registered in the PROSPERO Registry (CRD42022352164) [23, 25].

### Eligibility criteria

The literature selected for this study was based on the following criteria:

#### Study design

Randomised controlled trials (RCTs), controlled (non-randomised) clinical trials (CCTs), prospective and retrospective comparative cohort studies, case–control studies and case series were included in the review. Case reports and case series that did not report data on clinical and functional results were excluded.

#### Participants

The studies were conducted on skeletally mature patients treated for at least two anterior cruciate ligament (ACL) reconstructions.

#### Interventions

Studies that reported data on clinical, functional and radiological outcomes following multiple (≥ 2) ACL reconstructions. For ACL reconstruction, the surgical technique (the type of graft used, number of bundles, fixation technique and tensioning protocol) and the rehabilitation protocol were collected.

#### Types of outcome measures

The outcome measures extracted from the studies were the Lysholm score, Subjective International Knee Documentation Committee (IKDC), Marx Scores, Tegner, Marx Scores, Knee Injury and Osteoarthritis Outcome (KOOS) scores, radiological changes, rate of return to sports, complications, failures and/or revision surgery.

### Information sources and search

A systematic search for relevant literature was performed on the PubMed (MEDLINE), Scopus, EMBASE and Cochrane Library databases. The publication date was not considered an inclusion criterion. The search was carried out in July 2022. Two independent reviewers (RD and AM) assisted in conducting and validating the search. The following search terms were used in the title, abstract and keywords fields: “ACL” or “anterior cruciate ligament” AND “revision” or “multiple” or “repeat”.

### Data collection and analysis

#### Study selection

The retrieved articles were first screened by title, and if found relevant, were screened further by reading the abstract. Then, the content of the articles was evaluated for eligibility.

The authors have separately reviewed to reduce the risk of bias all the selected articles, references, as well as articles excluded from the study. In case of any disagreement between the reviewers, the senior investigator made the final decision. At the end of the process, further studies that might have been missed were manually searched by going through the reference lists of the included studies and relevant systematic reviews.

#### Data collection process

The data were extracted from the selected articles by the first two authors using a computerized tool created with Microsoft Access (Version 2010, Microsoft Corp, Redmond Washington). Each article was validated again by the first author before analysis. For each study, data from the patients was extracted (age, gender, duration between injury and surgery and follow-up evaluation), their injuries (type, aetiology and associated injuries), the surgical technique (the type of grafts used, the number of bundles, fixation technique, extra-articular procedures and tensioning protocol), the rehabilitation protocol, post-operative outcomes, rate of complications and the rate of return to sports.

#### Level of evidence

The Oxford Levels of Evidence set by the Oxford Centre for Evidence-Based Medicine was used to categorise the level of evidence [24].

#### Evaluation of the quality of studies

The quality of the selected studies was evaluated using the Methodological Index for Nonrandomized Studies (MINORS) score [27]. The checklist includes 12 items, out of which the last four are specific to comparative studies. Each item was given a score of 0–2 points. The ideal score was set at 16 points for non-comparative studies and 24 for comparative studies.

#### Statistical analysis

The extracted quantitative parameters (age, follow-up time and results of the PROMs) were given as mean ± standard deviation (SD) when provided in the articles. Otherwise, alternative values like median or range were extracted. Due to the high statistical and methodological heterogeneity in the included studies, a meta-analysis comparing the results between patients with and without concomitant surgeries was not possible. Instead, a narrative description and comparison of the clinical results were performed.

## Results

### Search results

**Fig. 1:**
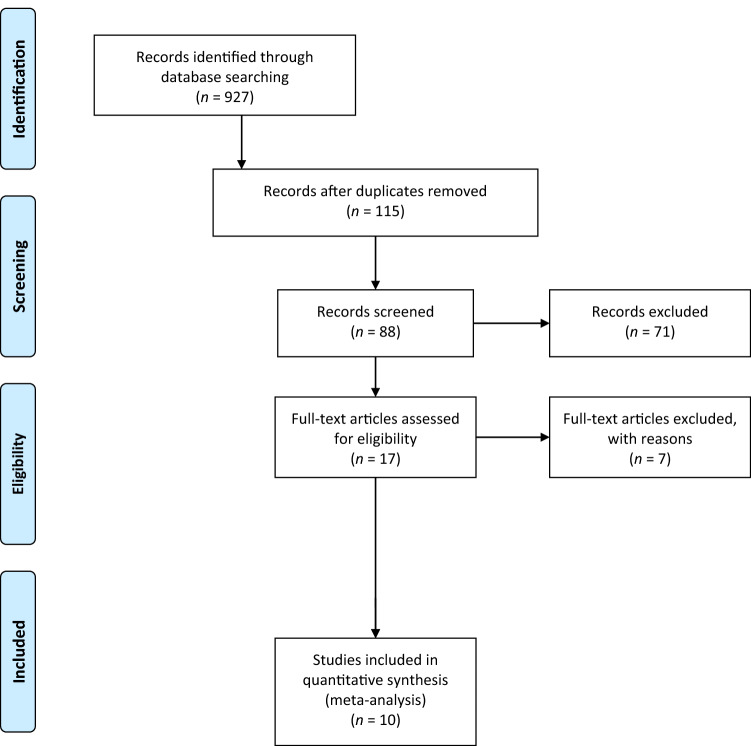
A flowchart of the literature screening performed in this study

The electronic search yielded 927 studies. The 812 duplicates were removed, leaving 115 studies, out of which 88 were excluded after reviewing the abstracts, with a final number of 27. An additional 17 articles were excluded based on the aforementioned inclusion and exclusion criteria [10]. This left 10 studies for analysis [1–3, 7, 9, 11, 13, 14, 31, 32]. Figure 1 shows the flowchart depicting the selection process for the studies. The analysed studies had a mean MINORS score of 13 (range 12–14), which confirmed the methodological quality of the available literature (Table 1).

**Table 1:** Characteristics of the selected studies

Authors, year	MINORS	Patients (*n*)	M:F	Age mean ± SD (range)	Follow-up (months)	Associated injuries
Yoon, 2019 [32]	13	20	15:5	33.8 ± 9.9 (22–55)	43.0 ± 24.1	7 medial meniscus tear 11 medial + lateral meniscus 1 MFC grade IV cartilage lesion 2 LFC grade IV cartilage lesion 2 trochlear groove grade IV cartilage lesion
Helito, 2022 [14]	12	6	3:3	28.5 ± 8.2	34.1 ± 12.8	2 medial meniscus tear 2 lateral meniscus tear
Ahmed, 2017 [1]	13	29	16:13	26.4 (14–54)	145.2 (39.6–230.4)	
Buda, 2013 [2]	13	24	24:0	30 (19–49)	39.6 (24–84)	10 medial meniscus tear 3 lateral meniscus tear 4 osteochondral lesions (2 grade III and 2 Grade IV)
Chen, 2013 [3]	14	151	93:58	29.6		62 medial meniscus tear 44 lateral meniscus tear 8 MFC cartilage lesion 32 MTP cartilage lesion 46 LFC cartilage lesion 28 LTP cartilage lesion 41 Trochlea cartilage lesion 59 patella cartilage lesion 6 MCL 2 LCL
Dini, 2019 [7]	14	17	12:5	28.4 (19–41)	29.6 (13–58)	5 medial meniscus tear 3 lateral mensicus tear 5 cartilage lesion
Engler, 2020 [9]	13	14	3:11	39.8 (21–58)	42 (24–79)	11 meniscal tear 5 chondral injury
Gorodischer, 2021 [11]	12	9	9:0	32 (30–34)	27 (24–39)	3 medial meniscus tear 1 lateral meniscus tear 4 cartilage lesion
Griffith, 2013 [13]	13	15	8:7	27 (18–57)	60 (24–120)	6 medial meniscus tear 1 lateral mensicus tear 1 combined medial and lateral meniscus tear 9 cartilage lesions
Wegrzyn, 2009 [31]	12	10	8:2	30 (17–48)	117 (54–168)	8 medial meniscus tear 3 lateral meniscus tear 2 bilateral meniscus tear 7 cartilage lesion

*MFC* medial femoral condyle, *MTP* medial tibial plateau, *LFC* lateral femoral condyle, *28 LTP* lateral tibial plateau, *MCL* medial collteral ligament, *LCL* lateral collateral ligament

### Patient characteristics

Table 1 shows the characteristics of the cohorts of the 10 selected studies and a summary of their data. The cohort consisted of 295 patients [191 (64.7%) men and 104 (35.3%) women] with a mean age of 29.9 ± 2.8 years (range 14–58 years). The mean postoperative follow-up (reported in all studies except one) was 66.9 ± 44.7 months (range 13–230.4 months).

Associated injuries were 103 (34.9%) medial meniscus tears, 57 (19.3%) lateral meniscus tears, 14 (4.7%) combined medial plus lateral meniscus tears, 11 (3.7%) meniscal tears (not specified), 252 (85.4%) cartilage lesion, 6 (2.0%) medial collateral ligament injury and 2 (0.7%) lateral collateral ligament injuries.

**Table 2:** Surgical and Rehabilitation Protocol

Lead author	Graft type	Fixation technique		Surgical technique	1 or 2 Stage	Bundle	Accessory procedures*	Tension protocol	Rehabilitation protocol		
		Femur	Tibia						Brace splint	Bearing	ROM
Yoon, 2019 [32]	18 allo ns 2 mixed			13 Transtibial 7 Transportal	One stage	Single			Yes	To tolerance from day 0	Restricted for 3 weeks
Helito, 2022 [14]	6 allo AT	Interfefence screw	Interference screw	Transportal	One stage	Single	6 ALL	30° of flexion	No	Partial WB as tolerated from day 0	Free from day 0
Ahmed, 2017 [1]	29 auto contro HT	Titanium screw	Titanium screw	Transportal	One stage	Single			No	Partial WB as tolerated from day 0	Free from day 0
Buda, 2013 [2]	9 allo AT 15 allo TP	1 Titanium staples	2 Titanium staples	Over the top	One stage	Single	24 extra-articular plasty	90° of flexion	Yes	Partial WB for the first 2 weeks	Passive from day 0
Chen, 2013 [3]	92 auto ns 54 allo ns 5 mixed										
Dini, 2019 [7]	5 auto homo PT 2 auto contro PT 1 auto contro HT 3 allo AT 5 allo TA 1 allo QS	Interference screw	Interference screw	Outside-in	13 Single 4 double	Single	12 extra-articular tenodesis	Yes	Yes	Partial WB for 3 weeks	0–90° for 4 weeks
Engler, 2020 [9]	8 allo PT 5 auto homo PT 1 auto homo HT	Interference screw	Interference screw	Transportal or transtibial	Single stage	Single			Yes	Partial WB for 6 weeks	Passive motion from day 0
Gorodischer, 2021 [11]	1 auto homo PT 4 allo PT 4 allo TA	Intereference screw	Interference screw		8 Single stage 1 double stage	Single	5 ALL	60 degrees of flexion	Yes	Full WB after 2 weeks	Passive from day 0
Griffith, 2013 [13]	12 allo PT 1 auto homo HT 1 auto contro HT 1 auto homo PT	13 interference screw 1 endobutton	13 interference screw 2 bicortical screw		13 Single stage 2 double stage	Single					
Wegrzyn, 2009 [31]	4 auto homo PT 5 auto contro PT 1 auto homo QS	Interference screw	Interference screw		9 single stage 1 double stage	Single	1 high tibial valgus osteotomy				

*Allo* allograft, *auto* autologous, *PT* patellar tendon, *HT* hamstrings, *TA* tibialis anterior, *TP* tibialis posterior, *homo* homolateral, *contro* controlateral, *ns* not specified, *WB* weight-bearing, *ALL* reconstruction

**Table 3:** Clinical and functional outcomes, complications, and return to sports and activity

Lead author	IKDC		Lysholm		Tegner		Marx score		KOOS		Laxity		Return to sport	Complications	Failures
	Pre	Post	Pre	Post	Pre	Post	Pre	Post	Pre	Post	Pre	Post			
Yoon, 2019 [32]	Subjective: 47.8 ± 11.6 (17.2–65.5)	Subjective: 58.9 ± 10.7 (41.4–78.2) *	54.7 ± 15.0 (21.0–85.0)	68.0 ± 11.8 (43.0–88.0)*	3.5 ± 1.4 (1.0–6.0)	4.0 ± 1.3 (1.0–6.0)					STSD: 10.1 ± 4.0 (014–16.5)	STSD: 7.1 ± 4.7 (0.2–19.6)*			6
Helito, 2022 [14]		Subjective: 79.1 ± 6.3		82.8 ± 5.1								KT-1000: 2.0 ± 1.1	5 (83.3%) returned to sports	4: 2 superficial infections 1 cyclops lesion 1 flexion loss	0
Ahmed, 2017 [1]		Subjective: 84										KT-1000: Anteroposterior laxity: 2.3 mm			8
Buda, 2013 [2]	Subjective: 40. ± 6 6.8	Subjectivr: 81.3 ± 14.0*										KT-1000: passive: 3.4 ± 0.7 mm KT -1000 active: 3.1 ± 1.1 mm	17 (71%) returned to their sports activity		
Chen, 2013 [3]								6.74 (0–16)							
Dini, 2019 [7]	Objective: 6 B 8 C 3D	Objective: 6 A* 8 B 3 C	43 ± 9	87 ± 7*							KT-1000: 5.8 ± 0.6	KT-1000: 1.5 ± 0.4*	4 (17%) to pre-injury level		
Engler, 2020 [9]		Subjective: 70 (41–95)			8.3 (3–10)	6.3 (5–9)									3
Gorodischer, 2021 [11]	Subjective: 57 (45–60)	Subjective: 77 (70–90)*	65(55–72)	90 (86–95)*						Pain: 93 (64–96) sympotms: 94 (83–97) ADL 96 (90–100) Sports 75 (50–90) QOL 50 (43–81)		KT-1000: 2 (1–8)mm	3 (33%) returned to same level		
Griffith, 2013 [13]	Subjective: 59	Subjective: 80*	60	82*	6.0	4.5									2
Wegrzyn, 2009 [31]		Objective: 2 A 5 B 2 C 1 D										KT-1000: 1.3 ± 1.9 mm (range, 0–6 mm	2 (20%) same level		

*IKDC* International Knee Documentation Committee, *KOOS* Knee Injury and Osteoarthritis Outcome Score, *STSD* side-to-side difference

*Statistical significant improvement

### Surgical protocol and rehabilitation protocol

All procedures were performed using a single bundle technique; a double-stage surgery for the revision had been performed only in 8 (2.7%) cases, and in all other cases, a single-stage revision was performed. The graft selected for the revision was an allograft in 139 (47.1%) cases [72 (24.4%) not specified, 18 (6.1%) Achilles tendon, 15 (5.1%) tibialis posterior, 9 (3.1%) tibialis anterior, 1 (0.3%) quadriceps and 24 (8.1%) patellar tendon]; in 111 (37.6%) cases, it was an autologous homolateral graft [92 (31.2%) not specified, 16 (5.4%) patellar tendon, 2 (0.7%) hamstring and 1 (0.3%) quadriceps]; in 38 (12.9%) cases, an autologous contralateral graft [31 (10.5%) hamstring and 7 (2.4%) patellar tendon], while in 7 (2.4%) cases, it was a mixed graft. For the femoral fixation, 7 studies reported the use of a screw, while 1 study reported the use of staples of the endobutton system. For tibial fixation, the use of a titanium screw was reported in all cases except for one in which the use of a staple was mentioned.

Five different techniques were reported for the re-revision of ACL reconstruction: transtibial in 2 studies, transportal in 4 studies, outside-in in 1 study and over the top in 1 study. All studies reported a single bundle technique.

An extra-articular plasty was performed in 47 (15.9%) patients with anterolateral ligament injury, 11 (3.7%) who underwent anterolateral ligament reconstruction, and in 24 (8.1%) patients the extra-articular plasty as planned in the over-the-top technique, and 12 (4.1%) received an extra-articular tenodesis with a modified Macintosh technique). Only in 1 (0.3%) case, a high tibial valgus osteotomy was performed. Only three studies reported degrees for tibial screw fixation, which ranged from 30° to 90°. Two studies did not suggest the use of a post-surgery brace, while it was recommended in 5. Weight-bearing was allowed from day 0 in 7 studies (Table 2).

### Clinical and functional outcomes

In all studies that reported pre-and post-operative IKDC (subjective and objective) and Lysholm scores, there was a significant improvement compared to the pre-operative value (*p* < 0.05). In detail, subjective IKDC ranged from IKDC_pre_ 48.7 ± 7.8 to 76.2 ± 8.9 (*p* < 0.05).

In pre-operative IKDC, there were 6 patients with grade B, 8 with grade C, and 3 with grade D, while in post-operative, there were 8 patients with grade A, 13 with grade B, 5 with grade C and 1 with grade D.

Lysholm score ranged from Lysholm_pre_ 54.3 ± 7.8 to Lysholm_post_ 80.2 ± 8.5. Tegner score ranged from Tegner_pre_ 5.6 ± 2.0 to Tegner_post_ 4.8 ± 1.0 (*p* < 0.05). KOOS and Marx’s Score were reported, respectively, only in 2 studies with only final follow-up values (KOOS: Pain: 93; Symptoms: 94; ADL: 96; Sports: 75; QOL: 50; Marx Score: 6.7) (Table 3).

### Laxity

In 2 studies, laxity was measured pre-and post-surgery, and there was a significant improvement in the stability of the knee (*p* < 0.05). At the final follow-up, laxity measured with KT-1000 resulted to be 2.2 ± 0.6 mm.

### Return to sports

A total of 31 (10.5%) out of 295 patients returned to their pre-injury activity level.

### Complications and failure

A total of 19 (6.4%) re-ruptures were found, while only in 4 cases (1.4%) complications (all minors) were reported, which were 2 (0.7%) superficial infections, 1 (0.3%) cyclops lesion and 1 (0.3%) flexion loss.

## Discussion

The most important findings of the current systematic review are that even after a re-revision ACL reconstruction (rrACLR), knee function was improved as reflected by the significant improvements in the Lysholm knee scores and both in the objective and subjective IKDC scores and the improved Tegner activity level, there was still a considerably low rate of return to pre-injury sports level activity.

The re-revision ACL surgery offers appreciable outcomes; however, the possibility of returning to sports activity is less when compared to those after revision ACL reconstructions. In the meta-analysis by Grassi et al., the authors reported that the overall return to any sporting activity was 84% and the rate of return to pre-injury level of activity was only 52% [12]. The rate of return to sports at the pre-injury level in the current study stands lower at 10.5%. The performance of the knee after re-revision (rrACLR) could not only be affected by the quality of the reconstruction but also by other adjunctive factors. Most importantly, the presence of concomitant injuries and the management of those would also negatively influence the outcome following a rrACLR. In the current study, the most common injuries present concomitantly were meniscal injuries, followed by cartilage lesions. The influence of the concomitant injuries on the knee function, following a re-revision ACLR, is not easy to quantify and hence becomes an intangible factor while considering this outcome.

Despite this poor return to sport, the knees concerning clinical examination for laxity, performed well after the re-revision surgery, with significant improvements in laxity as measured by KT-1000; however, this was reported only in 2 studies. Given that following rrACLR, the knee stability is quite improved, it would not be incorrect to assume that other factors are at play with respect to post-rrACLR sport participation.

An overwhelming majority of cases in the current review were carried out by a single-staged re-revision procedure, with only 2.7% of cases using a two-staged procedure. The surgical techniques used were heterogenous in our review. A previous meta-analysis by Colatruglio et al. on outcomes of one versus two stages revision of ACLR showed that the outcomes of both the single and double-stage techniques were comparable, even though there were certain indications, where a one-staged procedure was considered to be better. Strong evidence of the superiority of one technique over the other is lacking [4]. A study by Mitchell et al. also found no difference in the outcome following single- and two-staged revision ACL reconstructions [22]. Literature regarding the staging of procedures in the re-revision setting is also lacking. However, the current systematic review also demonstrates good outcomes following single-staged re-revision since the review has more single-staged revisions. The results were similar to the findings of Mitchell et al. and Colatruglio et al. Thus, the surgeons may prefer a single-staged re-revision procedure unless the specific situation warrants a two-staged revision procedure [4, 22].

Regarding graft choice, allograft was the most preferred in this review, and the most commonly used allograft was the Achilles tendon. The second most commonly used was a bone-patellar tendon-bone graft from the same limb. The least utilised were autografts from the opposite limb, for which hamstring tendons were commonly used and quadriceps. The ideal graft choice in the setting of a re-revision is yet to be clarified. The high utilisation of allografts in rrACLR can be accounted for by the fact that other graft options have been exhausted when a patient undergoes multiple revisions. A Systematic review on allograft ACLR by Mariscalco et al. studied autograft versus nonirradiated allograft [19]. Similarly, graft failure rate, laxity and patient-reported outcome scores were found in both groups [19]. Similar results were also observed by Cvetanovich et al. [6]. There have been reports of greater failure rates with allograft as compared to bone-patellar-tendon-bone autograft, possibly due to the longer duration of graft incorporation predisposing to non-traumatic ACL graft failures [6, 18, 30]. The graft of choice for the re-revision of ACL reconstruction and the use of allograft are areas that need further study in the future.

Out of 295 of rrACLR, only 48 cases reported extra-articular procedures or a tibial osteotomy; in current literature, there is a growing interest in additional procedures like these to increase the knee’s stability after an ACL reconstruction, especially in re-revision settings.

Several authors confirmed that an excessive posterior tibial slope (PTS) increases tensions within the ACL and exacerbates the risk of injury [33]. The normal PTS is within the range of 5°–7°, depending on the measurement technique, and is considered pathologic if it exceeds 12° [33]. For revision ACL reconstructions, however, surgeons must consider the correction of an excessive PTS, especially after the failure of two or more consecutive procedures [33].

The potential benefit of combining ALLR with ACLR is greater rotational stability, which leads to a decrease in both the high failure rates seen particularly in young patients and progressive osteoarthritic changes seen after isolated ACLR [34].

Further study is warranted to compare directly HTO and ALL reconstruction alongside multiple-revision ACL reconstruction versus ACL reconstruction alone [34].

**Fig. 2:**
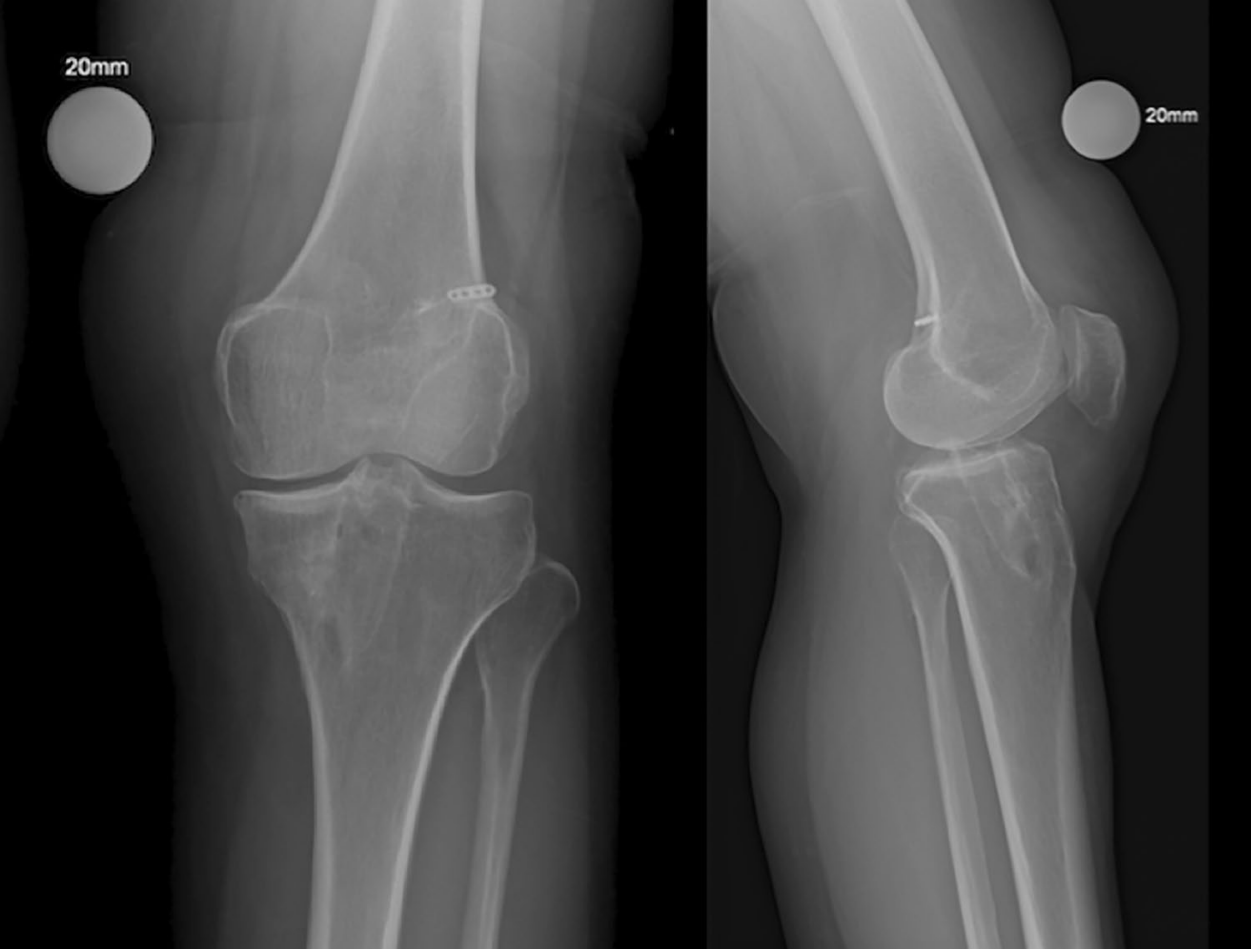
Pre-operative anteroposterior and lateral X-ray of a re-revision surgery case; left knee. The widened and increased tibial tunnel and probable mobilisation of the femoral plateau are visible

**Fig. 3:**
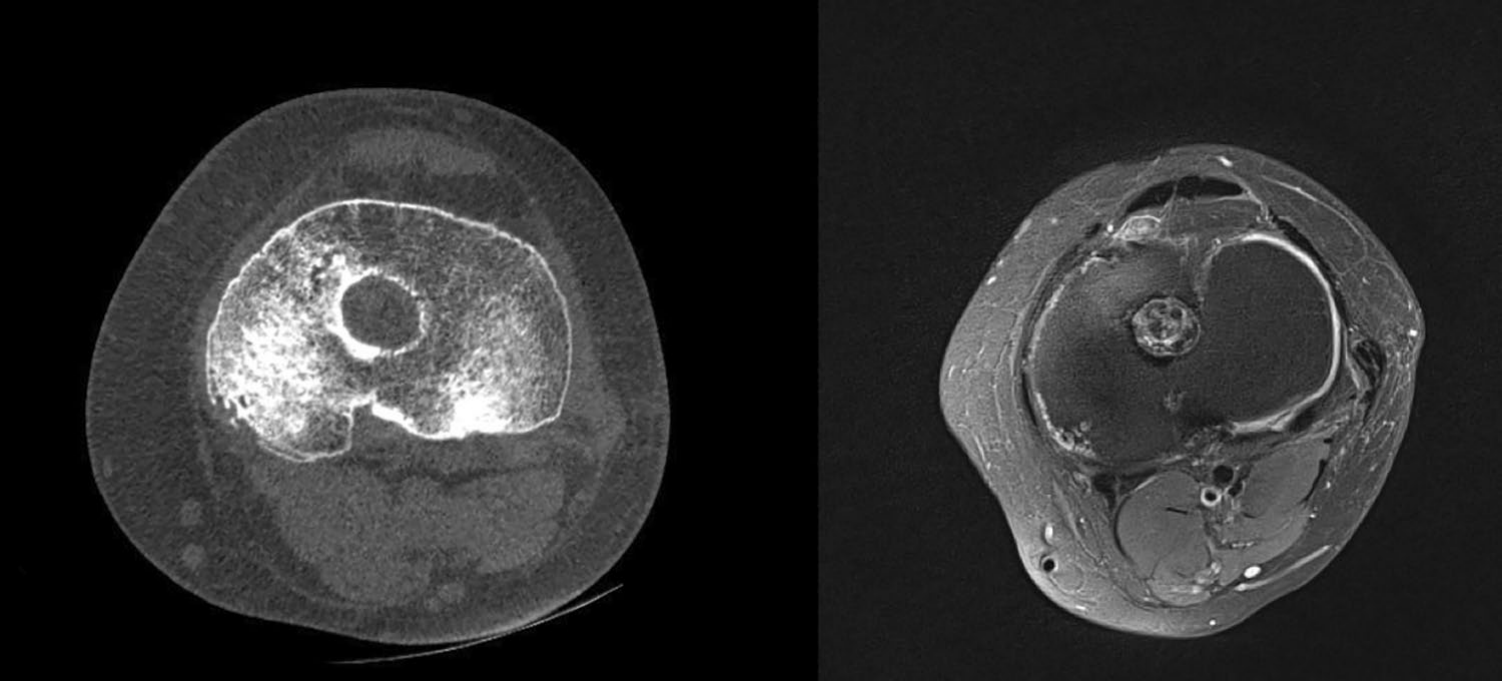
Pre-operative axial CT-Scan (on the left) and MRI (on the right) of a re-revision surgery case showing an enlargement of the previous tibial tunnel

**Fig. 4:**
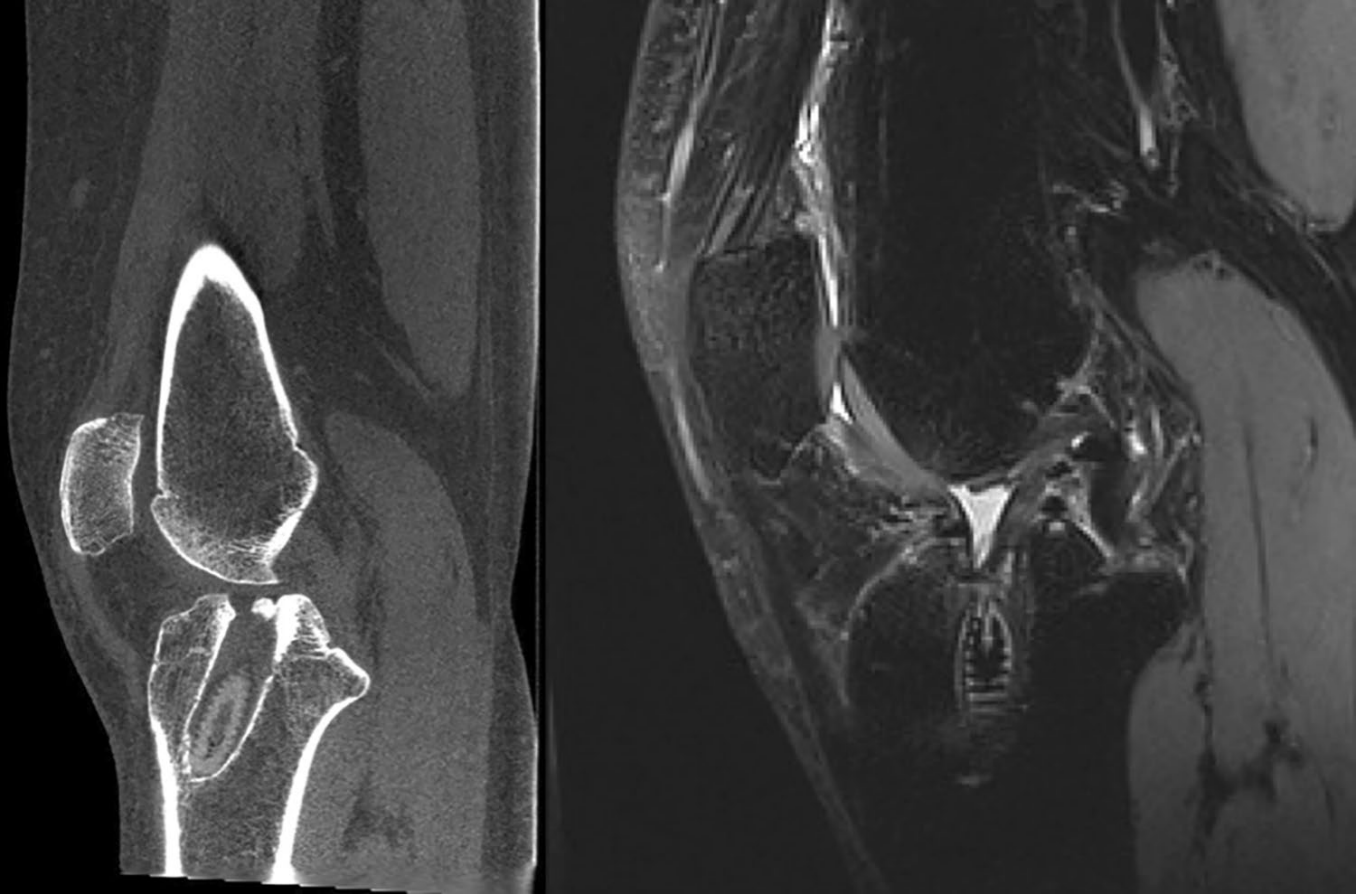
Pre-operative sagittal CT-Scan (on the left) and MRI (on the right) of a re-revision surgery case showing enlargement of the previous tibial tunnel

**Fig. 5:**
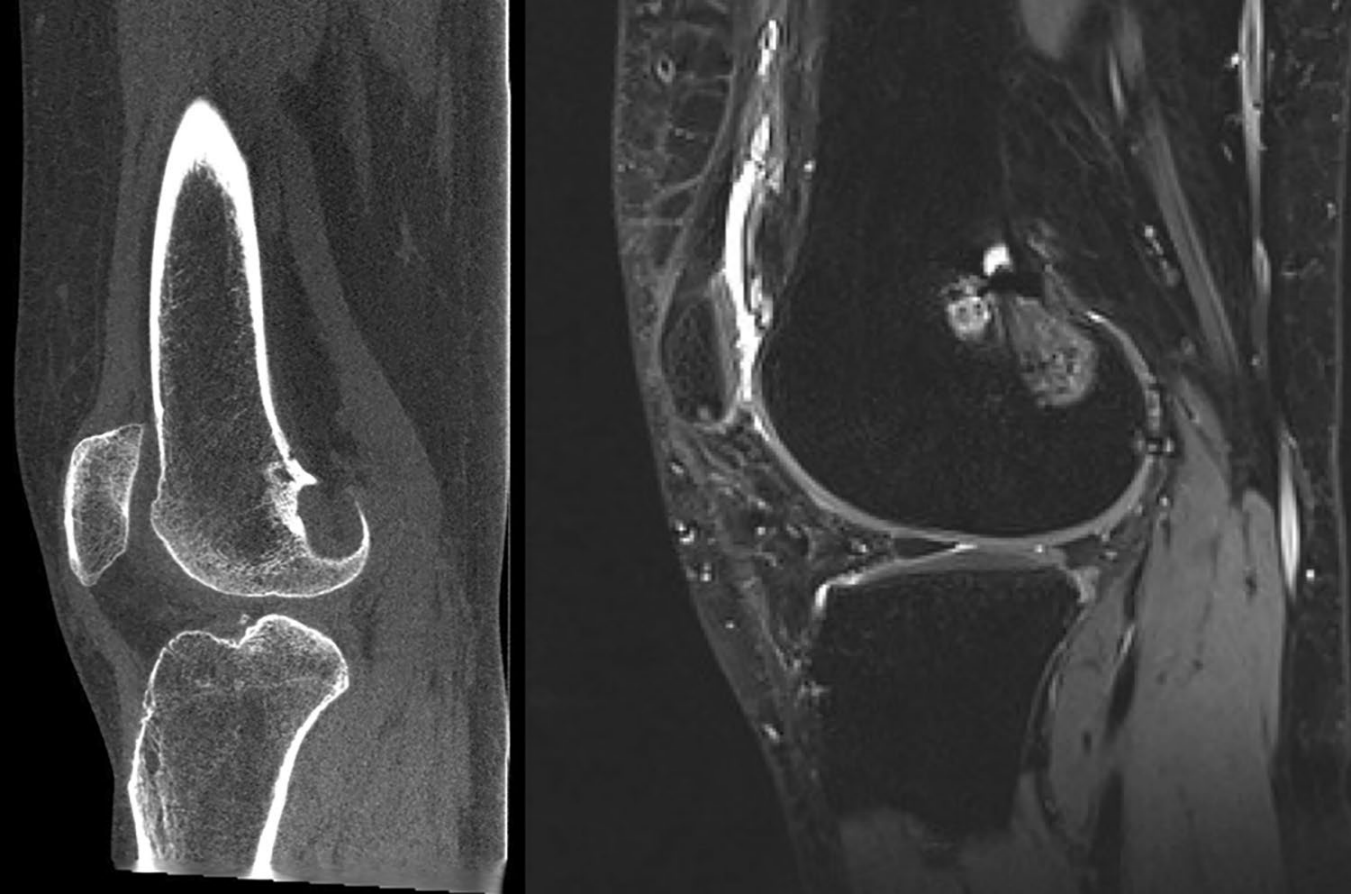
Pre-operative sagittal CT-Scan (on the left) and MRI (on the right) of a re-revision surgery case showing bone resorption on the femoral neo-ACL insertion

The overall complication rate was low and the ones observed were all minor. Hence, the re-revision of ACL reconstruction can be considered a safe and effective procedure with good outcomes, irrespective of whether it is performed single-staged or two-staged or whether it utilises both autografts and allografts (Figs. 2, 3, 4, and 5).

To the author’s best knowledge, this is one of the first systematic reviews on the re-revision of ACL reconstruction. However, the review was limited by the heterogeneous reporting of subjective and objective outcomes in the available studies. Also, it is difficult to consider the outcomes as those of re-revision ACL in isolation as they were concomitant in injuries in most of the cases with additional procedures to the knee. There were no control groups and no comparative analysis could be done.

## Conclusions

Multiple revisions of anterior cruciate ligament reconstruction allow acceptable clinical results and a good degree of knee stability with a low rate of subsequent new re-ruptures but a scarce level of return to pre-injury sports activity; whenever possible, it is preferred to revise the ligament in one stage. This surgery remains a big challenge for orthopaedic surgeons and many doubts remain regarding the ideal grafts, additional extra-articular procedures and techniques to use to prevent another rupture.

## Supplementary Information

Below is the link to the electronic supplementary material.

Supplementary file1 (PDF 1882 KB)
